# Smoke-Free Policies and Smoking Cessation in the United States, 2003–2015

**DOI:** 10.3390/ijerph16173200

**Published:** 2019-09-02

**Authors:** Andrea R. Titus, Lucie Kalousova, Rafael Meza, David T. Levy, James F. Thrasher, Michael R. Elliott, Paula M. Lantz, Nancy L. Fleischer

**Affiliations:** 1 Department of Epidemiology, School of Public Health, University of Michigan, Ann Arbor, MI 48109, USA (R.M.) (N.L.F.); 2 Department of Sociology, University of California-Riverside, Riverside, CA 92521, USA; 3 Lombardi Comprehensive Cancer Center, Georgetown University, Washington, DC 20007, USA; 4 Department of Health Promotion, Education, and Behavior, Arnold School of Public Health, University of South Carolina, Columbia, SC 29208, USA; 5 Department of Biostatistics, School of Public Health, University of Michigan, Ann Arbor, MI 48109, USA; 6 Gerald R. Ford School of Public Policy, University of Michigan, Ann Arbor, MI 48109, USA

**Keywords:** tobacco control policies, impact analysis, policies reducing disparities in tobacco use, policy impacts on vulnerable populations

## Abstract

(1) Background: Smoking restrictions have been shown to be associated with reduced smoking, but there are a number of gaps in the literature surrounding the relationship between smoke-free policies and cessation, including the extent to which this association may be modified by sociodemographic characteristics. (2) Methods: We analyzed data from the Tobacco Use Supplement to the Current Population Survey, 2003–2015, to explore whether multiple measures of smoking restrictions were associated with cessation across population subgroups. We examined area-based measures of exposure to smoke-free laws, as well as self-reported exposure to workplace smoke-free policies. We used age-stratified, fixed effects logistic regression models to assess the impact of each smoke-free measure on 90-day cessation. Effect modification by gender, education, family income, and race/ethnicity was examined using interaction terms. (3) Results: Coverage by workplace smoke-free laws and self-reported workplace smoke-free policies was associated with higher odds of cessation among respondents ages 40–54. Family income modified the association between smoke-free workplace laws and cessation for women ages 25–39 (the change in the probability of cessation associated with coverage was most pronounced among lower-income women). (4) Conclusions: Heterogeneous associations between policies and cessation suggest that smoke-free policies may have important implications for health equity.

## 1. Introduction

Despite decades of progress in curbing tobacco use, smoking remains a leading cause of morbidity and mortality in the United States [1]. Moreover, smoking and smoking-related illnesses are increasingly characterized by socio-demographic disparities. In 2017, the prevalence of smoking was 23.1% among adults with less than a high school education, compared to 7.1% among adults with an undergraduate degree [1]. The expansion of policies that effectively encourage smoking cessation, particularly among vulnerable groups, is urgently needed.

Smoking restrictions, including laws and policies that limit smoking in workplaces, restaurants, and bars, have been extensively studied as tools to reduce smoking [2,3] and increase smoking cessation [4,5,6]. For example, a review by Hopkins et al. found that exposure to smoke-free policies among workers was associated with a median increase in successful tobacco use cessation of 6.4 percentage points (interquartile interval= 2.0 p.p. to 9.7 p.p) [5]. However much of the evidence on the effectiveness of smoking restrictions on cessation is drawn from studies of earlier time periods, such as the 1990s and early 2000s, and it is not clear that these results remain relevant today [5]. More recently published studies on smoking restrictions have found mixed results with regard to the effects of these policies on quit attempts [7,8] and several aspects of the contemporary relationship between smoking restrictions and cessation remain understudied. For example, it is not known whether workplace smoke-free laws passed at multiple jurisdictional levels remain effective in the midst of the rapid proliferation of employer-based voluntary smoke-free policies. It is also unclear how smoking restrictions interact with the landscape of smoking disparities. Analyses focusing on the differential effects of smoking restrictions on an array of smoking outcomes have yielded inconsistent results [8,9,10] and several reviews have noted that the equity impacts of smoke-free laws remain mixed or inconclusive [11,12,13].

In this study, we examined whether smoking restrictions in workplaces and hospitality venues were associated with cessation between 2003 and 2015 in nationally representative data. We utilized multiple measures of smoking restrictions that encompass both regulatory policies (smoke-free laws) and smoke-free policies from other sources (e.g., employer-based policies). We also assessed the potential impact of smoking restrictions on health disparities by systematically examining whether each smoke-free measure was differentially associated with smoking cessation by gender, education, race/ethnicity, and income, and the intersection of these variables with age.

## 2. Materials and Methods 

### 2.1. Study Population

We used four waves of cross-sectional data from the U.S. Tobacco Use Supplement to the Current Population Survey (TUS-CPS), spanning 2003–2015. The CPS uses a multi-stage sampling design to yield a sample that is representative of the civilian, non-institutionalized adult population at the state and national levels [14]. The TUS is administered alongside the CPS in select years and months and contains detailed questions on tobacco use. All eligible individuals within a selected household are interviewed [14]. Data were downloaded from the Integrated Public Use Microdata Series (IPUMS) at the University of Minnesota [15]. The Census Bureau imputed missing data on age, race/ethnicity, and gender for all waves and missing data on family income for the last two waves [16]. Remaining missing income data (approximately 10% of respondents in first two waves) were multiply imputed using the method of chained equations with IVEware version 0.3 [17]. Variables used in the imputation included year, age, race/ethnicity, education, gender, marital status, family size, employment status, and longitudinally ascertained family income observations.

### 2.2. Analytic Sample

The analytic sample consisted of current smokers and former smokers who reported quitting within the past year. In order to limit our study to established smokers, we excluded individuals if we did not have information on their smoking behavior twelve months earlier and they reported an age of initiation into smoking “fairly regularly” within two years of their current age. We also limited the sample to respondents between the ages of 25 and 65. We chose this age range to capture individuals who were likely to have completed their educational attainment (lower limit of age 25) and who were of working age (upper limit of age 65). We excluded data from proxy respondents. Analyses incorporated self-response survey weights. 

### 2.3. Smoking Cessation

The outcome measure was a binary variable representing 90-day smoking cessation. The outcome was considered present if an individual in the analytic sample reported quitting smoking at least 90 days prior to the survey. The threshold of 90 days was chosen to mitigate concerns about relapse, which would be more pronounced with 30-day and 60-day cessation measures.

### 2.4. Smoke-Free Laws and Self-Reported Workplace Smoke-Free Policies

We explored multiple measures of exposure to smoking restrictions. Continuous variables capturing smoke-free laws in workplaces and hospitality venues (restaurants or bars) were created to represent the percent of the population covered by each type of law within each U.S. core-based statistical area (CBSA), comprised of single or multiple counties with economic and social linkages and a “core” population center of at least 10,000 people [18]. Information on smoke-free laws was obtained from the American Nonsmokers’ Rights Foundation (ANRF) Tobacco Control Laws Database [19]. Laws were considered present only if they met criteria for being considered “100% smoke-free” by the ANRF. Based on methods described in previous literature [20], data on smoke-free laws passed at the city, county, and state-level were combined with Census Bureau population estimates [21] to calculate the percent of each CBSA’s population covered by smoke-free laws for each month and year that the TUS-CPS survey was administered. Approximately one-third of the analytic sample (unweighted) did not reside within a CBSA. These respondents were assigned a state-specific measure representing the number of individuals outside of CBSAs covered by smoke-free laws divided by the entire non-CBSA population within each state. Two separate variables were created to represent smoke-free law coverage of 1) workplaces and 2) hospitality venues (restaurants or bars). Restaurant and/or bar law coverage was combined into a single variable due to high correlation between these law types.

In addition to CBSA-level measures of smoke-free law coverage, we also constructed a survey-based binary variable to represent whether each individual reported being covered by a workplace smoke-free policy, regardless of whether the policy was instituted by the government, an employer, or another source. A workplace policy was considered present if an individual reported that smoking was “not allowed in any work areas” and “not allowed in any public areas” when asked to describe smoking policies at their place of work. These questions were only asked of individuals who were not self-employed or retired and who reported working within the past week in an indoor environment that was not someone’s home, so the analytic sample for this exposure was limited to this subsample of survey respondents. 

### 2.5. Key Sociodemographic Variables

We explored interactions with four primary sociodemographic variables to evaluate the relationship between each smoke-free measure and cessation disparities: Gender (male, or female), education (less than high school, high school or equivalent, some college, or college or above), race/ethnicity (non-Hispanic White (NHW), non-Hispanic Black (NHB), Hispanic, or other non-Hispanic), and annual family income (<$15k; $15k–$29,999; $30k–$49,999; $50k–$74,999; or >$75k).

### 2.6. Covariates

In order to examine interactions with age, we used age-stratified models (25–39; 40–54; and 55–65). We chose these age categories based on observed trends in smoking and cessation. We included a continuous age variable in all models to control for residual confounding by age. To adjust for other aspects of the tobacco control environment, we included a variable representing the state-level annual average cost of a cigarette pack from the CDC’s “Tax Burden on Tobacco, Volume 51”, [22] and a variable representing state-level annual tobacco control expenditures per capita, based on information from the Campaign for Tobacco-Free Kids [23]. Both of these variables were adjusted for inflation, using 2016 as the reference year. Finally, we controlled for state-level anti-smoking sentiment by including two variables representing the weighted proportion of individuals in each state 1) reporting that they believed that smoking should not be allowed in bars and 2) indicating that no one was allowed to smoke in their home. These variables were derived from self-response data in each survey period of the TUS-CPS. Models assessing the relationship between smoke-free workplace laws and self-reported smoke-free workplace policies were adjusted for law coverage in hospitality venues, in addition to the covariates previously described. When the exposure was smoke-free law coverage of hospitality venues, models controlled for smoke-free law coverage of workplaces. 

### 2.7. Statistical Analysis

Separate, age-stratified logistic regression models were used to examine the relationship between each type of smoke-free measure and smoking cessation, with fixed effects for year and state to control for time trends and unmeasured time-invariant state-level contextual factors. We used the full analytic sample to evaluate the relationships between smoke-free laws in workplaces and hospitality venues and cessation. A restricted analytic sample including only individuals who were asked about smoke-free workplace policies in the TUS was used to evaluate the relationship between self-reported policies and cessation. We explored differential associations between each smoke-free variable and gender, education, race/ethnicity, and income through the inclusion of interaction terms in separate models. In Model 1, we examined bivariate associations between each covariate and smoking cessation, with state and year fixed effects. In Models 2 and 3, we explored the adjusted relationship between smoke-free laws or self-reported workplace policies and cessation without interaction terms. For each exposure, we then ran models that included an interaction term between the exposure and gender. Next, we ran models that included an interaction between the exposure and an additional sociodemographic variable (education, race/ethnicity, and family income), and a set of models with a triple interaction term between the exposure, gender, and the additional sociodemographic variable to test pre-specified questions regarding the effectiveness of smoke-free policies by the sociodemographic factors and their intersections with gender. We tested the overall significance of each two-way and three-way interaction. Statistical significance of three-way interaction terms suggested potential effect modification by a combination of variables, which we then explored in more detail by running gender-stratified models. Finally, we adjusted our effect modification analysis for multiple comparisons using the Benjamini–Hochberg correction method. We calculated critical thresholds for *p*-values for each exposure within each age strata, using a false discovery rate threshold of 0.05 [24]. We plotted predicted probabilities of cessation when interactions were statistically significant after adjusting for multiple comparisons.

We conducted several sensitivity analyses, including stratifying our analysis of workplace smoke-free laws by working status (employed vs. not); using law exposure variables with a one-year time lag; and examining whether there were differential effects for laws passed within the previous two years, compared to laws in place for more than two years. We also tested whether the inclusion of additional variables representing state-level demographic measures (i.e., percent below the federal poverty level and percent unemployed) would impact results. Finally, we examined the sensitivity of our results to the inclusion of variables capturing e-cigarette and smokeless tobacco use. All analyses were conducted in Stata SE, version 14.0 and incorporated survey weights.

## 3. Results

### 3.1. Sample Characteristics

Details of the analytic sample are included in Table 1. The total number of individuals in the sample was 102,834. Ninety-day smoking cessation increased over the study period, from 6.4% in 2003 to 8.9% in 2014–2015. Average smoke-free law coverage in 2003 was 9.7% for workplace laws and 17.2% for laws in hospitality venues, rising to 63.8% and 72.4% for workplace and hospitality laws, respectively, in 2014–2015. Among individuals who were asked about smoke-free workplace policy coverage, 72.1% reported that smoking was not allowed in work or public areas, increasing from 69.8% in 2003 to 74.7% in 2014–2015.

### 3.2. Association Between Smoke-Free Measures and Cessation

The results of bivariate models and adjusted models without interaction terms are reported in Table 2. In adjusted models without interaction terms, living in a CBSA with 100% workplace law coverage was associated with a higher odds of smoking cessation for individuals ages 40–54 (odds ratio (OR) = 1.27; 95% confidence interval (CI) = 1.00, 1.62), compared to living in a CBSA with no coverage. This OR corresponded to an average increase in the probability of cessation of 1.4 percentage points. Coverage by a self-reported workplace policy was also associated with a higher odds of cessation for individuals ages 40–54 (OR = 1.22; 95% CI = 1.02, 1.45). The average increase in the probability of cessation associated with working in an environment with a smoke-free policy was 1.5 percentage points. Neither workplace smoke-free laws nor self-reported workplace policies were associated with cessation for individuals aged 25–39 or 55–65. There were no statistically significant associations between smoke-free hospitality law coverage and cessation for any age group in adjusted models without interaction terms.

### 3.3. Effect Modification by Sociodemographic Factors

*p*-values for all models with two-way and three-way interaction terms are summarized in Table 3. We did not find any statistically significant interactions in two-way interaction models exploring interactions between each policy exposure and each sociodemographic characteristic (gender, education, race/ethnicity, or income) after adjusting for multiple testing. The three-way interaction between workplace smoke-free laws, gender, and family income was statistically significant for the youngest age group (*p*-interaction_law×gender×income_ = 0.004), as was the three-way interaction between hospitality smoke-free laws, gender, and education for the middle age group (*p*-interaction_law×gender×education_ = 0.039). After applying the Benjamini–Hochberg correction method, only the *p*-value for the interaction between workplace laws, gender, and income remained below the critical threshold. Detailed results from all model specifications including interaction terms are available in the Supplementary Material.

**Figure 1:**
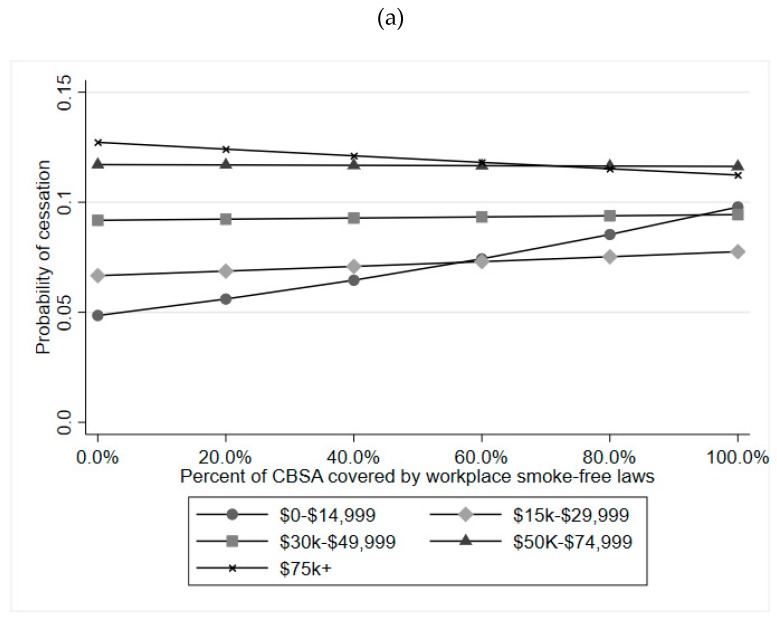

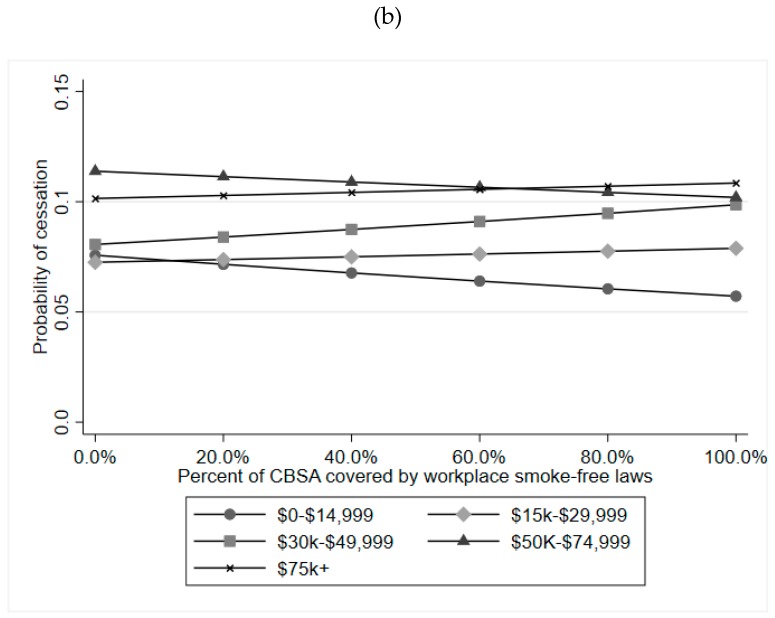
Predicted probability of cessation associated with workplace smoke-free law coverage by family income for (**a**) females and (**b**) males.

To further examine the significant interaction between workplace laws, gender, and family income, we limited the sample to adults ages 25–39 and included a two-way interaction term between workplace laws and family income in models stratified by gender. We found evidence of effect modification by income for females (*p*-interaction_law×income_ = 0.002), but not for males (*p*-interaction_law×income_ = 0.234). Figure 1 contains marginal predicted probability plots for cessation associated with smoke-free law coverage of workplaces, across categories of family income, for both men and women between the ages of 25 and 39. The change in cessation associated with smoke-free law coverage was most pronounced among women with annual family incomes <$15k. Within this income group, the probability of cessation for women living in a CBSA with full workplace smoke-free law coverage was approximately twice that of women in CBSAs with no coverage. Workplace law coverage did not appear strongly associated with cessation at any other income level.

### 3.4. Sensitivity Analyses

When we examined whether the associations between cessation and workplace smoke-free laws differed if the sample was restricted only to working adults, we found that the results were similar in magnitude and direction, although no relationships reached statistical significance at the 0.05 level, likely due to reduced sample size. We also examined whether results using smoke-free law variables that were lagged by one year were consistent with results from non-lagged variables and found that associations were similar across the two specifications. We ran models using smoke-free law variables with three levels based on the timing of the law’s passage (law passed within two years prior to the survey, law passed more than two years prior to the survey, no law passed). We found no evidence that the timing of the laws’ passage resulted in differential associations with cessation. The inclusion of variables capturing smokeless tobacco and e-cigarette use slightly attenuated associations. These variables were not included in final models because they were hypothesized to potentially mediate the relationship between smoking restrictions and cessation. Finally, additional state-level demographic variables representing the percent of the population below the federal poverty level and the percent of the population that was unemployed did not impact results and so were not included in final models.

## 4. Discussion

In this study, we examined the association between several measures of smoking restrictions and cessation, and we included a systematic analysis of differential associations across population subgroups. We found that both CBSA-level workplace law coverage and self-reported workplace policies were associated with a higher odds of 90-day cessation for individuals ages 40–54; we did not find that smoking restrictions were associated with cessation across other age groups. The point estimates for workplace smoking restrictions were in line with a review of smoke-free policies that reported a range of ORs for cessation associated with exposure to smoke-free policies of 1.21–1.92 [5].

That we did not find more consistent associations between any smoke-free measure and cessation across all age groups may be somewhat surprising, given that several evidence reviews have reported positive associations between smoking restrictions and cessation [4,5,25]. One potential explanation is that these reviews are based on data from earlier time periods, compared to the timespan of data included in this analysis, and that the effects of smoking restrictions may have decreased over time. The tobacco control landscape has changed considerably in recent decades, including the expansion of voluntary smoke-free policies, as well as other tobacco control interventions, including media campaigns and taxes on tobacco products [26]. More recent analyses of smoke-free laws and quit attempts have yielded mixed results. An evaluation of 2009 data from the National Health Interview Survey did not find an association between smoke-free laws and quit attempts [7], while an analysis of longitudinal data from the Coronary Artery Risk Development in Young Adults (CARDIA) study found that smoking bans in bars and restaurants were associated with increased quit attempts, although these results were sensitive to the functional form of control variables representing secular time trends [8]. Neither of the analyses explored cessation as an outcome.

The evaluation of different measures of smoke-free workplace coverage is a strength of our study. The two workplace variables captured different dimensions of smoke-free work environments, as well as potentially different mechanisms through which smoking bans might impact smoking behavior. The self-reported measure represented exposure to both smoke-free laws and policies from other sources (e.g., an employer) and encompassed the relationship between smoking restrictions and cessation among individuals who worked in environments that would be directly impacted by these policies. With the CBSA-based variable, all individuals were “exposed” or “unexposed” to smoke-free laws in their local area, regardless of employment status. In addition to capturing associations among working people, using the area-level exposure allowed for smoke-free laws to impact cessation through changing broader community norms around smoking behavior. Examining associations among all individuals may be important to determining population-level effects, as prior research has noted that the full impact of smoking restrictions is difficult if not impossible to disentangle from other factors associated with the implementation of such restrictions, including local media coverage and outreach [6].

In our sample, it is also interesting to note that while there was a substantial increase in smoke-free workplace laws over the time period of our study (9.7%–63.8%), the increase in the proportion of respondents indicating coverage by workplace smoking restrictions began at a relatively high level and increased only modestly (69.8%–74.7%). It is likely that the impact of expanded smoke-free workplace laws in this study may have been muted by pre-existing policies put in place by private firms. The lack of concordance between the change in the ecologic variable and the change in the self-reported variable over the study period further underscores the importance of considering multiple approaches to measuring exposure to smoking restrictions.

In our analysis of effect modification, the association between workplace smoke-free laws and smoking cessation among women aged 25–39 was strongest in the lowest income group (<$15k annual family income), compared to women at other income levels. This finding may be partially explained if lower income women were less likely to be covered by employer-instituted smoke-free policies compared to higher income women, and thus may have been more likely to be directly impacted by expanded smoke-free law coverage. Prior research using TUS-CPS data from 1998–2002 (before the widespread implementation of smoke-free laws) found that women with lower income levels were less likely to report smoke-free policies in their workplaces, compared to women with higher incomes [9]. A study using data from the 2014–2015 wave of the TUS-CPS also found that the proportion of workers reporting coverage by a 100% smoke-free policy was lowest among individuals with an annual household income <$35,000, compared to individuals at higher income levels [27]. An avenue for future research could be to investigate whether the impact of legislated smoking restrictions on cessation is stronger in contexts where voluntary policies are less prevalent, as in many countries that rapidly adopted policies recommended by the Framework Convention on Tobacco Control (FCTC).

Aside from age and the intersection of age, income, and gender, we found limited evidence of differential associations across population subgroups. There was no evidence of effect modification by race/ethnicity. Education also did not appear to modify relationships between any smoke-free measure and cessation, although there was evidence of joint effect modification by education and gender for hospitality smoke-free laws. However, this finding did not remain significant after adjusting for multiple testing. The null findings for these effect modification variables are consistent with a number of prior studies, primarily focused on smoking prevalence and intensity [12,28,29]. In a review, Thomas et al. found insufficient evidence that the effects of smoking restrictions in workplaces and public places varied by income, education, or ethnicity. Effects by age were inconsistent, and there was no evidence that associations differed by gender [12]. The perceived equity impact of smoking restrictions may also be highly sensitive to the outcome being studied. For example, in their analysis of longitudinal CARDIA data, Mayne et al. found that the relationship between hospitality smoke-free law coverage and current smoking was stronger among individuals of higher SES, but that the association between hospitality smoke-free laws and quit attempts was stronger among lower-SES individuals [8]. Our findings regarding effect modification by income suggest that there may be a stronger association between workplace smoke-free laws and cessation among women with lower SES, although these results should be interpreted with caution given the limitations described below, including the use of cross-sectional data in this analysis. Future research should examine this potential relationship in a longitudinal sample.

This study has a number of limitations. First, while we accounted for state-level tobacco control expenditures and average cigarette prices, we were not able to control for all tobacco control interventions, including media campaigns and sub-state level variation in tobacco taxes. Similarly, we did not account for whether there may have been partial smoking bans in place that did not meet the criteria for being characterized as 100% smoke-free. Our measures also did not consider compliance, although prior studies in the U.S. have suggested that compliance to comprehensive smoking bans tends to be high [30]. However, if the extent of compliance differed across sociodemographic groups, this could have implications for the differential effectiveness of smoke-free policies. This is an avenue for future research. Exposure to smoke-free laws and policies was likely misclassified for some individuals. Using a 90-day cessation measure, we were not able to test for permanent cessation. As our data were cross-sectional, we were not able to observe longitudinal changes in smoking behavior and the extent to which these coincided with implementation of smoking restrictions, which limits the causal assertions we can make about their impact on smoking cessation. Although all effect modification analyses were pre-specified and we applied a correction method for multiple testing, it is possible that significant findings were due to chance, given the number of model specifications included in this analysis. A substantial body of literature has elaborated on the potential for spurious results associated with multiple comparisons [31,32]. In addition, we did not consider the extent to which smoke-free policies may have been introduced alongside other cessation support services, particularly in workplaces [27]. While outside of the scope of this study, such cessation services, including counseling and self-help materials, may impact the relationship between smoke-free policies and cessation. They may also be increasingly important to consider given incentives under the Affordable Care Act to expand employer-based tobacco cessation programs [33]. Finally, we did not examine relationships between smoke-free laws and other aspects of smoking behavior, such as smoking initiation or intensity, or explore additional populations characterized by health disparities, including individuals with mental illness or those living in rural environments. These are areas for future research.

Strengths of this study include pooling data from multiple waves of a nationally representative survey that provided a large sample to explore differential effects of newly implemented smoke-free laws and policies on cessation across several key sociodemographic variables. Our approach to systematically evaluating associations between smoking restrictions and cessation across population subgroups can be adapted to evaluate other tobacco control policies, although the mechanisms of health equity impacts may be different for other interventions. We also used a detailed measure of smoke-free law coverage, which assigned individuals a probability of coverage based on all smoke-free laws passed at the state, county, and city level within each respondent’s CBSA of residence. We are not aware of any previous studies that have developed multiple measures of exposure to smoking restrictions—drawing on self-reports of workplace policies as well as information on the geographic distribution of smoke-free law coverage—to examine the relationship between these measures and disparities in smoking cessation. While our analysis is specific to the adult population in the U.S., the methods described here can be adapted to other contexts and countries that have expanded smoking restrictions in recent years.

## 5. Conclusions

We found that workplace smoking restrictions were inconsistently associated with 90-day smoking cessation, and that results varied by age, income, and gender. We did not find evidence that smoke-free laws in hospitality venues were associated with cessation. Our study updates evidence from prior decades on the impacts of smoke-free policies and underscores the importance of considering health equity in policy evaluations.

## Figures and Tables

**Table 1:** Weighted characteristics of the U.S. Tobacco Use Supplement to the Current Population Survey (TUS-CPS) analytic sample, 2003–2015.

	Overall	2003	2006–2007	2010–2011	2014–2015
Total *N*	102,834	28,026	28,807	25,302	20,699
Age Categories					
% 25–39	39.4%	40.4%	39.4%	38.8%	39.0%
% 40–54	41.0%	43.4%	42.5%	40.6%	36.6%
% 55–65	19.6%	16.1%	18.0%	20.5%	24.4%
Age (mean and s.d.)	43.4 (7.3)	42.6 (7.4)	43.1 (7.2)	43.6 (7.3)	44.3 (7.3)
% Male	54.2%	54.4%	54.2%	54.2%	54.2%
Race/Ethnicity					
% Non-Hispanic White	73.6%	74.5%	75.0%	73.4%	70.8%
% Non-Hispanic Black	11.5%	11.2%	10.7%	11.7%	12.4%
% Hispanic	9.7%	9.3%	9.4%	9.6%	10.8%
% Other Non-Hispanic	5.2%	5.1%	4.8%	5.2%	6.0%
Education					
% Less than High School	15.9%	16.2%	16.4%	15.2%	15.6%
% HS Graduate	38.5%	39.0%	38.8%	38.9%	37.3%
% Some College	31.0%	29.7%	30.0%	31.7%	32.9%
% College+	14.6%	15.1%	14.8%	14.2%	14.2%
Income					
% $0–14,999	19.4%	20.0%	17.2%	19.9%	20.9%
% $15,000–29,999	20.2%	20.8%	19.3%	21.3%	19.6%
% $30,000–49,999	23.7%	24.0%	24.5%	23.3%	22.6%
% $50,000–74,999	18.3%	18.5%	20.0%	17.3%	17.4%
% $75,000+	18.4%	16.8%	19.0%	18.2%	19.5%
% Recent Cessation (90-days)	7.4%	6.4%	7.2%	7.4%	8.9%
Average % CBSA coverage–workplace laws	40.3%	9.7%	29.6%	60.6%	63.8%
Average % CBSA coverage–hospitality laws	48.5%	17.2%	38.4%	68.6%	72.4%
Average % self-reported–workplace policies ^a^	72.1%	69.8%	67.0%	77.8%	74.7%

^a^ Proportion calculated among individuals who were asked about workplace policies within the TUS-CPS (*N* = 45,291).

**Table 2:** Odds ratios for smoking cessation associated with smoke-free laws and self-reported workplace policy coverage in adjusted models.

		Model 1 ^a^	Model 2 ^b^	Model 3 ^c^
Smoke-free workplace law	Ages 25–39	1.20 * (1.03–1.40)	1.06 (0.86–1.32)	
	Ages 40–54	1.12 (0.94–1.33)	1.27 * (1.00–1.62)	
	Ages 55–65	1.19 (0.95–1.50)	0.98 (0.71–1.34)	
Smoke-free hospitality law	Ages 25–39	1.20 * (1.02–1.40)	1.07 (0.86–1.34)	
	Ages 40–54	0.99 (0.83–1.19)	0.85 (0.66–1.10)	
	Ages 55–65	1.30 * (1.03–1.63)	1.26 (0.91–1.75)	
Smoke-free workplace policy (self-report)	Ages 25–39	1.05 (0.93–1.19)		1.00 (0.88–1.13)
	Ages 40–54	1.30 ** (1.10–1.55)		1.22 * (1.02–1.45)
	Ages 55–65	0.84 (0.65–1.09)		0.80 (0.62–1.04)

^a^ Bivariate associations, with state and year fixed effects. ^b^ Model controls for other smoke-free laws, education, race/ethnicity, family income, gender, age, state-level tobacco price, state-level anti-smoking sentiment, and state tobacco control expenditures, with state and year fixed effects. ^c^ Model controls for smoke-free laws in hospitality venues, education, race/ethnicity, family income, gender, age, state-level tobacco price, state-level anti-smoking sentiment, and state tobacco control expenditures, with state and year fixed effects. * *p* < 0.05; ** *p* < 0.01.

**Table 3:** *p*-values associated with interaction terms across age-stratified models ^a^.

	Ages 25–39	Ages 40–54	Ages 55–65
Workplace law interactions			
Gender	0.717	0.553	0.965
Education	0.969	0.712	0.815
Education×gender	0.412	0.230	0.378
Race/ethnicity	0.223	0.989	0.530
Race/ethnicity×gender	0.680	0.202	0.688
Family income	0.264	0.898	0.758
Family income×gender	0.004 ^†^	0.846	0.427
Hospitality law interactions			
Gender	0.934	0.940	0.539
Education	0.161	0.782	0.849
Education×gender	0.727	0.039	0.557
Race/ethnicity	0.234	0.377	0.054
Race/ethnicity×gender	0.643	0.477	0.611
Family income	0.284	0.207	0.374
Family income×gender	0.184	0.483	0.441
Self-reported workplace policy interactions			
Gender	0.922	0.751	0.810
Education	0.861	0.722	0.569
Education×gender	0.913	0.114	0.224
Race/ethnicity	0.155	0.934	0.578
Race/ethnicity×gender	0.581	0.456	0.601
Family income	0.888	0.278	0.553
Family income×gender	0.964	0.810	0.279

^a^ Each *p*-value represents a separate model. ^†^ *p*-value below the critical threshold after adjusting for multiple comparisons.

## Supplementary Materials

The following are available online at https://www.mdpi.com/1660-4601/16/17/3200/s1, Table S1: Odds ratios for cessation associated with smoke-free workplace law coverage by gender, Table S2: Odds ratios for cessation associated with smoke-free workplace law coverage across levels of education, and education and gender, Table S3: Odds ratios for cessation associated with smoke-free workplace law coverage across categories of race/ethnicity, and race/ethnicity and gender, Table S4: Odds ratios for cessation associated with smoke-free workplace law coverage across levels of family income, and family income and gender, Table S5: Gender-stratified odds ratios for cessation associated with smoke-free workplace law coverage across categories of family income, ages 25–39, Table S6: Odds ratios for cessation associated with smoke-free hospitality law coverage by gender, Table S7: Odds ratios for cessation associated with smoke-free hospitality law coverage across levels of education, and education and gender, Table, S8: Odds ratios for cessation associated with smoke-free hospitality law coverage across categories of race/ethnicity, and race/ethnicity and gender, Table S9: Odds ratios for cessation associated with smoke-free hospitality law coverage across levels of family income, and family income and gender, Table S10: Odds ratios for cessation associated with self-reported workplace smoke-free policy coverage by gender, Table S11: Odds ratios for cessation associated with self-reported workplace smoke-free policy coverage across levels of education, and education and gender, Table S12: Odds ratios for cessation associated with self-reported workplace smoke-free policy coverage across categories of race/ethnicity, and race/ethnicity and gender, Table S13: Odds ratios for cessation associated with self-reported workplace smoke-free policy coverage across levels of family income, and family income and gender.
