# Supplementary material for: Smoke-Free Policies and Smoking Cessation in the United States, 2003–2015
Source: Int J Environ Res Public Health. 2019 Sep 2;16(17):3200. doi: 10.3390/ijerph16173200 (PMC6747670; doi:10.3390/ijerph16173200)
Supplement: Supplementary file 1 [file ijerph-16-03200-s001.pdf]

S1. Odds ratios for cessation associated with smoke-free workplace law coverage by gender.

	Model 4		
	Ages 25–39	Ages 40–54	Ages 55–65
Smoke-free workplace law	1.04 (0.83–1.32)	1.23 (0.95–1.60)	0.97 (0.69–1.37)
Male	0.92 (0.82–1.04)	0.96 (0.85–1.09)	0.95 (0.80–1.13)
Law*Male	1.04 (0.86–1.25)	1.07 (0.86–1.32)	1.01 (0.77–1.32)

Model controls for other smoke-free laws, education, race/ethnicity, family income, gender, age, state-level tobacco price, state-level anti-smoking sentiment, and state tobacco control expenditures, with state and year fixed effects. **P*<0.05; ***P*<0.01; ****P*<0.001.

S2. Odds ratios for cessation associated with smoke-free workplace law coverage across levels of education, and education and gender.

	Model 5						Model 6					
	**Ages 25–39**	p_{int}	**Ages 40–54**	p_{int}	**Ages 55–65**	p_{int}	**Ages 25–39**	p_{int}	**Ages 40–54**	p_{int}	Ages 55–65	p_{int}
Smoke-free workplace law	1.04 (0.79–1.35)		1.20 (0.86–1.66)		1.07 (0.68–1.67)		0.91 (0.66–1.26)		1.28 (0.86–1.92)		1.22 (0.68–2.20)	
Male	0.94 (0.86–1.02)		0.99 (0.90–1.09)		0.95 (0.84–1.08)		0.84 (0.67–1.06)		1.01 (0.75–1.35)		1.23 (0.79–1.91)	
Education (College+ reference)												
Less than high school (HS)	0.34*** (0.27–0.43)		0.53*** (0.41–0.68)		0.76 (0.55–1.03)		0.30*** (0.21–0.42)		0.59** (0.41–0.83)		0.87 (0.54–1.38)	
HS or equivalent	0.45*** (0.38–0.53)		0.65*** (0.54–0.78)		0.82 (0.62–1.07)		0.43*** (0.34–0.53)		0.63*** (0.49–0.82)		0.97 (0.65–1.44)	
Some college	0.67*** (0.57–0.78)		0.83* (0.69–1.00)		0.94 (0.72–1.23)		0.62*** (0.51–0.76)		0.87 (0.68–1.13)		1.18 (0.79–1.75)	
Law×Education												
Law×Less than HS	1.10 (0.74–1.64)		1.09 (0.72–1.65)		0.79 (0.48–1.32)		1.60 (0.92–2.76)		0.72 (0.39–1.30)		0.87 (0.41–1.84)	
Law×HS or equiv.	1.03 (0.80–1.33)	0.969	1.15 (0.85–1.56)	0.712	0.94 (0.63–1.42)	0.815	1.15 (0.80–1.66)		1.14 (0.75–1.73)		0.83 (0.46–1.49)	
Law×Some college	1.02 (0.80–1.30)		1.01 (0.74–1.37)		0.89 (0.59–1.33)		1.17 (0.83–1.63)		0.86 (0.57–1.31)		0.67 (0.37–1.21)	
Law×Male							1.27 (0.87–1.84)		0.88 (0.53–1.44)		0.81 (0.41–1.58)	
Education×Male												
Less than HS×Male							1.25 (0.79–1.96)		0.82 (0.51–1.34)		0.80 (0.44–1.47)	
HS or equivalent×Male							1.10 (0.81–1.49)		1.04 (0.72–1.49)		0.75 (0.44–1.27)	
Some college×Male							1.13 (0.84–1.53)		0.90 (0.62–1.30)		0.68 (0.40–1.17)	
Law×Education×Male												
Law*Less than HS×Male							0.52 (0.24–1.13)		2.05 (0.90–4.68)		0.80 (0.29–2.22)	
Law*HS or equiv×Male							0.81 (0.49–1.35)	0.412	1.03 (0.56–1.88)	0.230	1.24 (0.55–2.77)	0.378
Law*Some college×Male							0.78 (0.48–1.27)		1.37 (0.74–2.53)		1.64 (0.73–3.70)	

Models control for other smoke-free laws, race/ethnicity, family income, gender, age, state-level tobacco price, state-level anti-smoking sentiment, state-level tobacco expenditures, and include state and year fixed effects. **P*<0.05; ***P*<0.01; ****P*<0.001.

S3. Odds ratios for cessation associated with smoke-free workplace law coverage across categories of race/ethnicity, and race/ethnicity and gender.

	Model 7						Model 8					
	Ages 25–39	p_{int}	Ages 40–54	p_{int}	Ages 55–65	p_{int}	Ages 25–39	p_{int}	Ages 40–54	p_{int}	Ages 55–65	p_{int}
Smoke-free workplace law	1.10 (0.89–1.37)		1.27 (0.99–1.62)		0.98 (0.71–1.35)		1.09 (0.86–1.38)		1.24 (0.95–1.63)		0.98 (0.69–1.39)	
Male	0.94 (0.86–1.02)		0.99 (0.90–1.09)		0.95 (0.84–1.08)		0.97 (0.86–1.11)		1.00 (0.87–1.15)		1.00 (0.83–1.21)	
Race/ethnicity (NHW reference)												
Non-Hispanic Black (NHB)	0.79 (0.61–1.01)		0.81 (0.63–1.03)		0.89 (0.65–1.21)		0.97 (0.71–1.33)		0.90 (0.66–1.24)		0.92 (0.60–1.40)	
Hispanic	1.34** (1.09–1.65)		1.30 (0.99–1.71)		1.00 (0.63–1.58)		1.41* (1.05–1.89)		1.67** (1.16–2.42)		1.25 (0.64–2.43)	
Other Non-Hispanic (NH)	0.91 (0.70–1.20)		0.76 (0.54–1.05)		0.84 (0.52–1.37)		1.11 (0.76–1.62)		0.48* (0.27–0.84)		1.29 (0.66–2.55)	
Law×Race/ethnicity												
Law×NHB	1.15 (0.75–1.76)		1.07 (0.72–1.59)		1.02 (0.62–1.68)		1.03 (0.60–1.78)		0.86 (0.50–1.49)		1.17 (0.59–2.30)	
Law×Hispanic	0.90 (0.64–1.26)	0.223	1.02 (0.68–1.52)	0.989	0.72 (0.34–1.53)	0.530	1.01 (0.63–1.63)		0.86 (0.49–1.52)		0.68 (0.23–2.05)	
Law×Other NH	0.65 (0.42–1.02)		0.98 (0.57–1.69)		1.54 (0.76–3.14)		0.51* (0.26–0.98)		1.84 (0.77–4.42)		1.02 (0.38–2.76)	
Law×Male							1.03 (0.84–1.27)		1.05 (0.83–1.32)		1.00 (0.75–1.34)	
Race/ethnicity×Male												
NHB×Male							0.65 (0.39–1.07)		0.80 (0.50–1.28)		0.93 (0.52–1.69)	
Hispanic×Male							0.92 (0.62–1.36)		0.66 (0.41–1.08)		0.68 (0.29–1.62)	
Other NH×Male							0.73 (0.43–1.22)		1.92 (0.97–3.80)		0.46 (0.18–1.22)	
Law×Race/ethnicity×Male												
Law×NHB×Male							1.28 (0.55–2.97)		1.50 (0.68–3.28)		0.75 (0.28–2.04)	
Law×Hispanic×Male							0.83 (0.43–1.59)	0.680	1.29 (0.59–2.81)	0.202	1.07 (0.24–4.74)	0.688
Law×Other NH×Male							1.49 (0.62–3.57)		0.38 (0.13–1.14)		2.08 (0.53–8.23)	

Models control for other smoke-free laws, education, family income, gender, age, state-level tobacco price, state-level anti-smoking sentiment, state-level tobacco expenditures, and include state and year fixed effects. *P<0.05; **P<0.01; ***P<0.001.

S4. Odds ratios for cessation associated with smoke-free workplace law coverage across levels of family income, and family income and gender.

	Model 9						Model 10					
	Ages 25–39	p_{int}	Ages 40–54	p_{int}	Ages 55–65	p_{int}	Ages 25–39	p_{int}	Ages 40–54	p_{int}	Ages 55–65	p_{int}
Smoke-free workplace law	1.30 (0.92–1.84)		1.29 (0.91–1.84)		1.08 (0.70–1.66)		1.98** (1.33–2.96)		1.35 (0.88–2.07)		1.19 (0.71–2.00)	
Male	0.94 (0.86–1.02)		0.99 (0.90–1.09)		0.95 (0.84–1.08)		1.45* (1.04–2.03)		1.01 (0.72–1.42)		0.93 (0.62–1.40)	
Family income (<$15K reference)												
$15K–$29,999	1.16 (0.91–1.46)		0.97 (0.76–1.24)		1.25 (0.95–1.66)		1.37* (1.01–1.85)		1.14 (0.83–1.58)		1.30 (0.88–1.91)	
$30k–$49,999	1.45** (1.17–1.79)		1.10 (0.88–1.38)		1.26 (0.95–1.67)		1.94*** (1.47–2.57)		1.18 (0.87–1.59)		1.37 (0.95–1.98)	
$50k–$74,999	2.02*** (1.62–2.52)		1.45** (1.15–1.82)		1.28 (0.95–1.73)		2.52*** (1.89–3.38)		1.42* (1.05–1.93)		1.12 (0.74–1.70)	
$75K+	1.96*** (1.57–2.46)		1.69*** (1.35–2.12)		1.45* (1.07–1.96)		2.78*** (2.06–3.76)		1.63** (1.21–2.20)		1.30 (0.84–1.99)	
Law×Family income												
Law×15K–$29,999	0.86 (0.59–1.26)	0.264	1.09 (0.74–1.62)	0.898	0.91 (0.59–1.41)	0.758	0.55* (0.34–0.88)		0.88 (0.52–1.49)		0.79 (0.43–1.43)	
Law×$30K–$49,999	0.88 (0.62–1.24)		1.01 (0.70–1.46)		0.83 (0.54–1.27)		0.48** (0.31–0.74)		0.97 (0.59–1.62)		0.59 (0.33–1.06)	
Law×$50K–$74,999	0.71* (0.50–1.01)		0.91 (0.63–1.32)		1.03 (0.66–1.60)		0.46** (0.30–0.73)		0.86 (0.53–1.40)		1.05 (0.57–1.93)	
Law×75K+	0.75 (0.53–1.07)		0.96 (0.68–1.36)		0.81 (0.52–1.26)		0.40*** (0.26–0.64)		0.89 (0.55–1.43)		0.83 (0.44–1.59)	
Law×Male							0.39** (0.22–0.69)		0.91 (0.52–1.59)		0.81 (0.43–1.53)	
Family income×Male												
$15K–$29,999×Male							0.70 (0.45–1.09)		0.72 (0.45–1.16)		0.94 (0.53–1.64)	
$30K–$49,999×Male							0.56** (0.37–0.84)		0.88 (0.57–1.37)		0.84 (0.49–1.44)	
$50K–$74,999×Male							0.64* (0.43–0.96)		1.03 (0.67–1.59)		1.27 (0.70–2.29)	
$75K×Male							0.51** (0.33–0.77)		1.06 (0.70–1.62)		1.21 (0.68–2.16)	
Law×Family income×Male												
Law×$15K–$29,999×Male							2.72** (1.28–5.77)	0.004	1.55 (0.71–3.40)	0.846	1.35 (0.56–3.25)	0.427
Law×$30K–$49,999×Male							3.59*** (1.77–7.25)		1.09 (0.52–2.30)		1.95 (0.83–4.56)	
Law×$50K–$74,999×Male							2.61** (1.31–5.22)		1.12 (0.55–2.31)		1.00 (0.41–2.43)	
Law×$75K×Male							3.68*** (1.81–7.44)		1.18 (0.58–2.36)		1.00 (0.41–2.42)	

Models control for other smoke–free laws, education, race/ethnicity, gender, age, state–level tobacco price, state–level anti–smoking sentiment, state–level tobacco expenditures, with state and year fixed effects. *P<0.05; **P<0.01; ***P<0.001.

S5. Gender-stratified odds ratios for cessation associated with smoke-free workplace law coverage across categories of family income, ages 25–39.

	Males	p_{int}	Females	p_{int}
Smoke-free workplace law	0.74 (0.43–1.27)		2.15** (1.37–3.37)	
Family income (<$15K reference)				
$15K–$29,999	0.95 (0.68–1.33)		1.41* (1.04–1.91)	
$30k–$49,999	1.07 (0.79–1.46)		2.00*** (1.51–2.66)	
$50k–$74,999	1.58** (1.17–2.15)		2.65*** (1.96–3.56)	
$75K+	1.39* (1.01–1.91)		2.91*** (2.14–3.97)	
Law×Family income				
Law×$15K–$29,999	1.49 (0.81–2.73)	0.234	0.55* (0.34–0.88)	0.002
Law×$30K–$49,999	1.70 (0.98–2.96)		0.48** (0.31–0.75)	
Law×$50K–$74,999	1.20 (0.70–2.06)		0.46*** (0.29–0.72)	
Law×$75K	1.47 (0.84–2.55)		0.40*** (0.25–0.64)	

Models control for other smoke–free laws, race/ethnicity, family income, gender, age, state–level tobacco price, state–level anti–smoking sentiment, state–level tobacco expenditures, with state and year fixed effects. *P<0.05; **P<0.01; ***P<0.001.

S6. Odds ratios for cessation associated with smoke-free hospitality law coverage by gender.

	Model 11		
	Ages 25–39	Ages 40–54	Ages 55–65
Smoke-free hospitality law	1.08 (0.85–1.37)	0.85 (0.64–1.13)	1.32 (0.92–1.88)
Male	0.94 (0.83–1.07)	0.98 (0.86–1.13)	1.00 (0.83–1.20)
Law×Male	0.99 (0.83–1.19)	1.01 (0.82–1.24)	0.92 (0.71–1.20)

Model controls for other smoke-free laws, education, race/ethnicity, family income, gender, age, state-level tobacco price, state-level anti-smoking sentiment, and state tobacco control expenditures, with state and year fixed effects. *P<0.05; **P<0.01; ***P<0.001.

S7. Odds ratios for cessation associated with smoke-free hospitality law coverage across levels of education, and education and gender.

	Model 12						Model 13					
	Ages 25–39	p_{int}	Ages 40–54	p_{int}	Ages 55–65	p_{int}	Ages 25–39	p_{int}	Ages 40–54	p_{int}	Ages 55–65	p_{int}
Smoke-free hospitality law	0.92 (0.70–1.21)		0.80 (0.57–1.12)		1.40 (0.91–2.16)		0.85 (0.62–1.18)		1.00 (0.66–1.49)		1.76 (0.99–3.13)	
Male	0.94 (0.86–1.02)		0.99 (0.90–1.09)		0.95 (0.84–1.08)		0.87 (0.66–1.13)		1.19 (0.86–1.65)		1.38 (0.85–2.24)	
Education (College+ reference)												
Less than high school (HS)	0.29*** (0.22–0.38)		0.51*** (0.39–0.68)		0.77 (0.55–1.08)		0.27*** (0.18–0.39)		0.68* (0.46–0.98)		0.93 (0.56–1.54)	
HS or equivalent	0.40*** (0.33–0.48)		0.64*** (0.53–0.79)		0.84 (0.63–1.12)		0.38*** (0.30–0.48)		0.70* (0.53–0.93)		1.07 (0.70–1.65)	
Some college	0.62*** (0.53–0.74)		0.82 (0.67–1.01)		0.95 (0.71–1.28)		0.59*** (0.47–0.73)		0.93 (0.70–1.23)		1.21 (0.78–1.87)	
Law×Education												
Law×Less than HS	1.44* (1.00–2.09)		1.12 (0.75–1.67)		0.80 (0.49–1.31)		1.80* (1.06–3.08)		0.57 (0.32–1.01)		0.81 (0.39–1.67)	
Law×HS or equiv.	1.26 (0.98–1.61)	0.161	1.13 (0.84–1.51)	0.782	0.91 (0.61–1.36)	0.849	1.39 (0.97–1.97)		0.93 (0.62–1.39)		0.72 (0.41–1.29)	
Law×Some college	1.14 (0.90–1.44)		1.01 (0.75–1.36)		0.89 (0.60–1.32)		1.26 (0.91–1.75)		0.80 (0.53–1.19)		0.69 (0.38–1.24)	
Law×Male							1.15 (0.80–1.65)		0.67 (0.42–1.07)		0.69 (0.36–1.32)	
Education×Male												
Less than HS×Male							1.15 (0.69–1.93)		0.61 (0.35–1.04)		0.74 (0.38–1.43)	
HS or equivalen×Male							1.09 (0.77–1.52)		0.86 (0.58–1.27)		0.66 (0.37–1.18)	
Some college×Male							1.13 (0.81–1.57)		0.80 (0.54–1.20)		0.68 (0.38–1.22)	
Law×Education×Male												
Law×Less than HS×Male							0.68 (0.32–1.41)		3.21** (1.45–7.10)		0.95 (0.36–2.49)	
Law×HS or equiv×Male							0.84 (0.52–1.38)	0.727	1.44 (0.80–2.57)	0.039	1.46 (0.67–3.22)	0.557
Law×Some college×Male							0.82 (0.51–1.32)		1.56 (0.87–2.81)		1.51 (0.69–3.34)	

Models control for other smoke-free laws, race/ethnicity, family income, gender, age, state-level tobacco price, state-level anti-smoking sentiment, state-level tobacco expenditures, and include state and year fixed effects
**P*<0.05; ***P*<0.01; ****P*<0.001

S8. Odds ratios for cessation associated with smoke-free hospitality law coverage across categories of race/ethnicity, and race/ethnicity and gender.

	Model 14						Model 15					
	Ages 25–39	p_{int}	Ages 40–54	p_{int}	Ages 55–65	p_{int}	Ages 25–39	p_{int}	Ages 40–54	p_{int}	Ages 55–65	p_{int}
Smoke-free hospitality law	1.07 (0.85–1.35)		0.88 (0.67–1.14)		1.27 (0.91–1.78)		1.08 (0.84–1.39)		0.90 (0.67–1.20)		1.36 (0.94–1.96)	
Male	0.94 (0.86–1.02)		0.99 (0.90–1.09)		0.95 (0.84–1.08)		1.00 (0.87–1.14)		1.04 (0.90–1.21)		1.07 (0.88–1.31)	
Race/ethnicity (NHW reference)												
Non-Hispanic Black (NHB)	0.84 (0.64–1.10)		0.77 (0.59–1.00)		0.99 (0.72–1.36)		1.03 (0.74–1.45)		0.88 (0.62–1.25)		1.12 (0.72–1.74)	
Hispanic	1.15 (0.88–1.50)		1.57** (1.16–2.13)		1.04 (0.60–1.79)		1.17 (0.80–1.71)		2.22*** (1.50–3.29)		1.36 (0.63–2.92)	
Other Non-Hispanic (NH)	0.95 (0.70–1.28)		0.84 (0.56–1.24)		0.56* (0.32–0.98)		1.20 (0.81–1.79)		0.70 (0.36–1.35)		0.97 (0.46–2.06)	
Law×Race/ethnicity												
Law×NHB	0.98 (0.65–1.48)		1.18 (0.79–1.75)		0.82 (0.50–1.34)		0.90 (0.53–1.52)		0.95 (0.56–1.62)		0.78 (0.39–1.55)	
Law×Hispanic	1.17 (0.84–1.62)	0.234	0.76 (0.51–1.12)	0.377	0.75 (0.38–1.48)	0.054	1.30 (0.81–2.10)		0.57* (0.34–0.97)		0.65 (0.25–1.69)	
Law×Other NH	0.69 (0.46–1.04)		0.83 (0.49–1.42)		2.46* (1.20–5/08)		0.54* (0.29–0.99)		0.89 (0.37–2.15)		1.57 (0.56–4.38)	
Law×Male							0.98 (0.80–1.20)		0.96 (0.76–1.21)		0.89 (0.67–1.18)	
Race/ethnicity×Male												
NHB×Male							0.65 (0.40–1.12)		0.77 (0.46–1.30)		0.79 (0.42–1.46)	
Hispani×Male							0.96 (0.59–1.59)		0.55* (0.31–0.97)		0.61 (0.22–1.69)	
Other NH×Male							0.67 (0.38–1.19)		1.32 (0.59–2.95)		0.33* (0.11–0.98)	
Law×Race/ethnicity×Male												
Law×NHB×Male							1.25 (0.56–2.83)		1.49 (0.68–3.27)		1.08 (0.41–2.88)	
Law×Hispanic×Male							0.84 (0.44–1.60)	0.643	1.62 (0.76–3.44)	0.477	1.31 (0.35–4.87)	0.611
Law×Other NH×Male							1.53 (0.67–3.45)		0.88 (0.30–2.64)		2.57 (0.62–10.61)	

Models control for other smoke-free laws, education, family income, gender, age, state-level tobacco price, state-level anti-smoking sentiment, state-level tobacco expenditures, and include state and year fixed effects. *P<0.05; **P<0.01; ***P<0.001.

S9. Odds ratios for cessation associated with smoke-free hospitality law coverage across categories of family income, and family income and gender.

	Model 16						Model 17					
	Ages 25–39	p_{int}	Ages 40–54	p_{int}	Ages 55–65	p_{int}	Ages 25–39	p_{int}	Ages 40–54	p_{int}	Ages 55–65	p_{int}
Smoke-free hospitality law	1.34 (0.95–1.87)		0.89 (0.63–1.26)		1.51 (1.00–2.30)		1.70* (1.13–2.56)		0.90 (0.59–1.36)		1.76* (1.05–2.95)	
Male	0.94 (0.86–1.02)		0.99 (0.90–1.09)		0.95 (0.84–1.08)		1.27 (0.87–1.84)		0.98 (0.68–1.41)		1.00 (0.64–1.55)	
Family income (<$15K reference)												
$15K–$29,999	1.16 (0.90–1.49)		0.89 (0.68–1.17)		1.39* (1.02–1.88)		1.22 (0.87–1.69)		1.07 (0.76–1.51)		1.58* (1.04–2.38)	
$30k–$49,999	1.57*** (1.25–1.98)		1.13 (0.89–1.44)		1.33 (0.99–1.80)		1.91*** (1.41–2.59)		1.23 (0.89–1.69)		1.38 (0.92–2.07)	
$50k–$74,999	2.07*** (1.63–2.64)		1.51** (1.18–1.92)		1.29 (0.94–1.78)		2.39*** (1.74–3.29)		1.38 (0.99–1.92)		1.14 (0.72–1.80)	
$75K+	2.05*** (1.60–2.63)		1.80*** (1.42–2.29)		1.56** (1.12–2.16)		2.68*** (1.92–3.72)		1.62** (1.17–2.23)		1.34 (0.84–2.14)	
Law×Family income												
Law×15K–$29,999	0.90 (0.62–1.28)		1.25 (0.86–1.84)		0.76 (0.49–1.18)		0.75 (0.47–1.21)		1.02 (0.62–1.69)		0.56 (0.31–1.01)	
Law×$30K–$49,999	0.77 (0.56–1.07)	0.284	0.96 (0.67–1.37)	0.207	0.76 (0.50–1.16)	0.374	0.55** (0.36–0.85)		0.90 (0.56–1.46)		0.63 (0.36–1.13)	
Law×$50K–$74,999	0.73 (0.52–1.01)		0.85 (0.60–1.21)		1.01 (0.65–1.57)		0.58* (0.37–0.90)		0.93 (0.58–1.50)		1.00 (0.54–1.87)	
Law×$75K+	0.74 (0.53–1.04)		0.86 (0.62–1.20)		0.74 (0.47–1.15)		0.50** (0.32–0.80)		0.93 (0.59–1.45)		0.80 (0.42–1.51)	
Law×Male							0.60 (0.35–1.03)		0.99 (0.58–1.69)		0.73 (0.39–1.38)	
Family income×Male												
$15K–$29,999×Male							0.89 (0.55–1.44)		0.69 (0.41–1.16)		0.77 (0.42–1.41)	
$30K–$49,999×Male							0.68 (0.43–1.05)		0.85 (0.53–1.37)		0.93 (0.52–1.66)	
$50K–$74,999×Male							0.74 (0.47–1.16)		1.17 (0.74–1.87)		1.23 (0.65–2.34)	
$75K×Male							0.59* (0.37–0.94)		1.21 (0.77–1.92)		1.27 (0.67–2.40)	
Law×Family income×Male												
Law×$15K–$29,999×Male							1.45 (0.71–2.96)		1.53 (0.71–3.36)		1.89 (0.80–4.49)	
Law×$30K–$49,999×Male							1.99* (1.04–3.82)	0.184	1.14 (0.56–2.32)	0.483	1.45 (0.65–3.33)	0.441
Law×$50K–$74,999×Male							1.65 (0.86–3.18)		0.85 (0.42–1.70)		1.06 (0.44–2.57)	
Law×$75K×Male							2.16* (1.11–4.22)		0.89 (0.45–1.73)		0.93 (0.38–2.27)	

Models control for other smoke-free laws, education, race/ethnicity, gender, age, state-level tobacco price, state-level anti-smoking sentiment, state-level tobacco expenditures, with state and year fixed effects. *P<0.05; **P<0.01; ***P<0.001.

S10. Odds ratios for cessation associated with self-reported workplace smoke-free policy coverage by gender.

	**Model 18**		
	Ages 25–39	Ages 40–54	Ages 55–65
Smoke-free workplace policy (self-report)	1.04 (0.87–1.24)	1.22 (0.97–1.55)	0.79 (0.55–1.13)
Male	1.02 (0.83–1.26)	0.93 (0.69–1.25)	0.94 (0.60–1.48)
Policy×Male	1.01 (0.79–1.29)	1.06 (0.75–1.48)	1.07 (0.64–1.78)

Model controls for smoke–free hospitality laws, education, race/ethnicity, family income, gender, age, state–level tobacco price, state–level anti–smoking sentiment, and state tobacco control expenditures, with state and year fixed effects. **P*<0.05; ***P*<0.01; ****P*<0.001.

S11. Odds ratios for cessation associated with self-reported workplace smoke-free policy coverage across levels of education, and education and gender.

	Model 19						Model 20					
	Ages 25–39	p_{int}	Ages 40–54	p_{int}	Ages 55–65	p_{int}	Ages 25–39	p_{int}	Ages 40–54	p_{int}	Ages 55–65	p_{int}
Smoke-free workplace policy (self–report)	1.08 (0.85–1.36)		1.05 (0.73–1.50)		1.06 (0.59–1.92)		1.05 (0.74–1.47)		1.19 (0.72–1.96)		0.60 (0.25–1.42)	
Male	1.05 (0.94–1.17)		0.97 (0.84–1.12)		0.97 (0.77–1.22)		0.98 (0.65–1.47)		1.02 (0.54–1.92)		0.45 (0.16–1.30)	
Education (College+ reference)												
Less than high school (HS)	0.45** (0.28–0.70)		0.38** (0.20–0.73)		0.85 (0.37–1.93)		0.27** (0.12–0.61)		0.33* (0.13–0.85)		0.43 (0.11–1.64)	
HS or equivalent	0.53*** (0.40–0.70)		0.55** (0.37–0.83)		0.91 (0.47–1.76)		0.49** (0.32–0.74)		0.69 (0.40–1.21)		0.66 (0.26–1.65)	
Some college	0.68** (0.52–0.90)		0.65* (0.43–0.98)		1.05 (0.53–2.05)		0.69 (0.47–1.02)		0.59 (0.33–1.07)		0.59 (0.22–1.60)	
Policy×Education												
Policy×Less than HS	0.85 (0.48–1.49)		1.22 (0.58–2.60)		1.01 (0.39–2.62)		1.17 (0.46–2.98)		1.17 (0.40–3.45)		2.47 (0.54–11.29)	
Policy×HS or equiv.	0.88 (0.63–1.22)	0.861	1.13 (0.71–1.78)	0.722	0.68 (0.33–1.41)	0.569	0.87 (0.54–1.40)		0.73 (0.39–1.37)		1.19 (0.42–3.31)	
Policy×Some college	0.93 (0.68–1.28)		1.30 (0.82–2.06)		0.66 (0.31–1.38)		0.94 (0.60–1.46)		1.32 (0.69–2.51)		1.45 (0.48–4.37)	
Policy×Male							1.04 (0.65–1.66)		0.77 (0.38–1.57)		2.96 (0.92–9.54)	
Education×Male												
Less than HS×Male							2.00 (0.75–5.29)		1.30 (0.36–4.69)		3.56 (0.67–18.75)	
HS or equivalent×Male							1.12 (0.64–1.96)		0.65 (0.29–1.46)		1.82 (0.50–6.54)	
Some college×Male							0.96 (0.56–1.65)		1.19 (0.53–2.68)		2.90 (0.76–11.05)	
Policy×Education×Male												
Policy×Less than HS×Male							0.67 (0.20–2.19)		1.15 (0.26–5.14)		0.19 (0.03–1.41)	
Policy×HS or equiv×Male							1.03 (0.53–1.99)	0.913	2.44 (0.98–6.08)	0.114	0.34 (0.08–1.48)	0.224
Policy×Some college×Male							1.00 (0.53–1.86)		0.98 (0.39–2.43)		0.23 (0.05–1.03)	

Models control for smoke–free hospitality laws, race/ethnicity, family income, gender, age, state–level tobacco price, state–level anti–smoking sentiment, state–level tobacco expenditures, and include state and year fixed effects. **P*<0.05; ***P*<0.01; ****P*<0.001.

S12. Odds ratios for cessation associated with self-reported workplace smoke-free policy coverage across categories of race/ethnicity, and race/ethnicity and gender.

	**Model 21**						**Model 22**					
	**Ages 25–39**	**p_{int}**	**Ages 40–54**	**p_{int}**	**Ages 55–65**	**p_{int}**	**Ages 25–39**	**p_{int}**	**Ages 40–54**	p_{int}	Ages 55–65	p_{int}
Smoke-free workplace policy (self-report)	0.99 (0.86–1.14)		1.24* (1.03–1.51)		0.76 (0.58–1.01)		1.00 (0.82–1.21)		1.19 (0.93–1.54)		0.80 (0.53–1.19)	
Male	1.04 (0.93–1.17)		0.98 (0.84–1.12)		0.97 (0.77–1.22)		1.09 (0.86–1.37)		0.97 (0.69–1.35)		1.12 (0.69–1.83)	
Race/ethnicity (NHW reference)												
Non-Hispanic Black (NHB)	0.70 (0.41–1.17)		0.98 (0.55–1.75)		0.66 (0.28–1.53)		0.86 (0.43–1.73)		1.28 (0.60–2.72)		0.99 (0.36–2.78)	
Hispanic	1.47* (1.05–2.06)		1.58 (0.91–2.64)		0.49 (0.17–1.42)		1.38 (0.81–2.37)		1.34 (0.58–3.08)		0.51 (0.06–4.10)	
Other Non-Hispanic (NH)	0.50** (0.30–0.83)		0.62 (0.27–1.44)		0.92 (0.30–2.80)		0.78 (0.40–1.51)		0.61 (0.19–1.99)		2.59 (0.71–9.51)	
Policy×Race/ethnicity												
Policy×NHB	1.33 (0.73–2.39)		1.02 (0.54–1.93)		1.51 (0.58–3.94)		1.24 (0.56–2.73)		0.81 (0.35–1.88)		1.22 (0.38–3.90)	
Policy×Hispanic	0.78 (0.51–1.18)	0.155	0.82 (0.45–1.51)	0.934	1.90 (0.55–6.57)	0.578	0.85 (0.45–1.60)		1.24 (0.50–3.12)		1.56 (0.15–16.23)	
Policy×Other NH	1.60 (0.91–2.82)		0.93 (0.36–2.38)		0.70 (0.17–2.92)		1.03 (0.46–2.30)		1.28 (0.34–4.85)		0.34 (0.06–2.13)	
Policy×Male							0.99 (0.75–1.30)		1.10 (0.75–1.60)		0.93 (0.53–1.63)	
Race/ethnicity×Male												
NHB×Male							0.69 (0.25–1.90)		0.57 (0.18–1.80)		0.34 (0.06–1.92)	
Hispanic×Male							1.09 (0.55–2.16)		1.24 (0.43–3.56)		0.91 (0.08–10.18)	
Other NH×Male							0.45 (0.17–1.22)		1.03 (0.21–5.07)		0.08* (0.01–0.86)	
Policy×Race/ethnicity×Male												
Policy×NHB×Male							1.09 (0.34–3.49)		1.60 (0.44–5.82)		1.72 (0.23–13.01)	
Policy×Hispanic×Male							0.88 (0.38–2.04)	0.581	0.45 (0.13–1.55)	0.456	1.40 (0.09–22.30)	0.601
Policy×Other NH×Male							2.20 (0.69–6.95)		0.58 (0.09–3.62)		7.01 (0.35–140.55)	

Models control for smoke-free hospitality laws, education, family income, gender, age, state-level tobacco price, state-level anti-smoking sentiment, state-level tobacco expenditures, and include state and year fixed effects. *P<0.05; **P<0.01; ***P<0.001.

S13. Odds ratios for cessation associated with self-reported workplace smoke-free policy coverage across categories of family income, and family income and gender.

	Model 23						Model 24					
	Ages 25–39	p_{int}	Ages 40–54	p_{int}	Ages 55–65	p_{int}	Ages 25–39	p_{int}	Ages 40–54	p_{int}	Ages 55–65	p_{int}
Smoke-free workplace policy (self-report)	1.10 (0.68–1.78)		1.86 (0.95–3.63)		1.31 (0.49–3.51)		1.16 (0.59–2.27)		1.54 (0.71–3.34)		2.08 (0.47–9.13)	
Male	1.05 (0.93–1.17)		0.97 (0.84–1.12)		0.97 (0.77–1.22)		1.35 (0.61–3.00)		0.68 (0.19–2.48)		2.01 (0.34–11.82)	
Family income (<$15K reference)												
$15K–$29,999	1.28 (0.80–2.06)		1.31 (0.64–2.66)		1.20 (0.43–3.35)		1.34 (0.68–2.65)		1.35 (0.59–3.09)		1.99 (0.44–8.99)	
$30k–$49,999	1.72* (1.10–2.69)		1.65 (0.86–3.19)		1.45 (0.55–3.83)		1.92* (1.01–3.68)		1.33 (0.60–2.95)		2.95 (0.68–12858)	
$50k–$74,999	1.89** (1.20–2.99)		2.07* (1.08–3.98)		1.51 (0.57–4.03)		2.26* (1.19–4.29)		1.91 (0.87–4.20)		1.22 (0.24–6.27)	
$75K+	2.10** (1.31–3.37)		2.09* (1.09–4.01)		1.22 (0.44–3.36)		2.62** (1.34–5.11)		1.73 (0.79–3.86)		1.88 (0.37–9.43)	
Policy×Family income												
Policy×15K–$29,999	0.96 (0.54–1.70)		0.63 (0.28–1.45)		0.70 (0.22–2.26)		0.92 (0.41–2.04)		0.68 (0.26–1.80)		0.36 (0.07–1.90)	
Policy×$30K–$49,999	0.79 (0.46–1.35)	0.888	0.59 (0.27–1.25)	0.278	0.41 (0.13–1.25)	0.553	0.79 (0.37–1.68)		0.82 (0.32–2.09)		0.19* (0.04–0.97)	
Policy×$50K–$74,999	1.02 (0.60–1.75)		0.58 (0.27–1.22)		0.60 (0.20–1.83)		0.90 (0.43–1.91)		0.63 (0.26–1.56)		0.67 (0.12–3.91)	
Policy×$75K+	0.87 (0.51–1.51)		0.71 (0.34–1.49)		0.73 (0.24–2.26)		0.78 (0.36–1.69)		0.88 (0.36–2.19)		0.42 (0.07–2.33)	
Policy×Male							0.91 (0.35–2.41)		1.67 (0.38–7.25)		0.37 (0.05–2.93)	
Family income×Male												
$15K–$29,999×Male							0.89 (0.35–2.30)		0.98 (0.21–4.58)		0.36 (0.04–2.91)	
$30K–$49,999×Male							0.79 (0.32–1.93)		1.67 (0.39–7.10)		0.21 (0.03–1.55)	
$50K–$74,999×Male							0.71 (0.29–1.74)		1.30 (0.32–5.30)		1.18 (0.15–9.17)	
$75K×Male							0.67 (0.27–1.67)		1.54 (0.38–6.33)		0.44 (0.06–3.38)	
Policy*Family income×Male												
Policy×$15K–$29,999×Male							1.10 (0.34–3.51)		0.76 (0.13–4.52)		4.35 (0.37–51.73)	
Policy×$30K–$49,999×Male							0.98 (0.33–2.88)	0.964	0.45 (0.08–2.35)	0.810	5.38 (0.52–55.87)	0.279
Policy×$50K–$74,999×Male							1.25 (0.42–3.69)		0.72 (0.15–3.59)		1.15 (0.11–12.52)	
Policy×$75K×Male							1.19 (0.39–3.58)		0.58 (0.12–2.87)		3.24 (0.31–34.04)	

Models control for smoke-free hospitality laws, education, race/ethnicity, gender, age, state-level tobacco price, state-level anti-smoking sentiment, state-level tobacco expenditures, with state and year fixed effects. *P<0.05; **P<0.01; ***P<0.001.
